# Whole-exome sequencing reveals cumulative rare variant burden associated with susceptibility to anti-tuberculosis drug-induced liver injury in a Han Chinese cohort

**DOI:** 10.3389/fphar.2026.1952049

**Published:** 2026-10-12

**Authors:** Yang Hao, Xiaoqing Zhang, Daiyun Huang, Shengyin Qin, Yunxiao Lin, Jianwen Ruan, Lu Shen, Na Zhang, Zhenting Wang, Cong Huai, Jianquan Zhang

**Affiliations:** 1 Hepatobiliary and Pancreatic Surgery, Haikou Research Institute of Xiangya School of Medicine, Central South University, Haikou, China; 2 Bio-X Institutes, Key Laboratory for the Genetics of Developmental and Neuropsychiatric Disorders (Ministry of Education), Shanghai Jiao Tong University, Shanghai, China; 3 Department of Pharmacy, The International Peace Maternity and Child Health Hospital, School of Medicine, Shanghai Jiao Tong University, Shanghai, China; 4 Wisdom Lake Academy of Pharmacy, Xi’an Jiaotong-Liverpool University, Suzhou, China; 5 Department of Hepatology, Haikou Research Institute of Xiangya School of Medicine, Central South University, Haikou, China; 6 Department of Urology, Haikou Research Institute of Xiangya School of Medicine, Central South University, Haikou, China

**Keywords:** anti-tuberculosis drug-induced liver injury, genetic susceptibility, pharmacogenomics, rare variant burden, whole-exome sequencing

## Abstract

**Background:**

Anti-tuberculosis drug-induced liver injury (AT-DILI) complicates 2-28% of tuberculosis treatments, limiting therapy and occasionally causing acute liver failure. Inter-individual susceptibility remains incompletely explained by known genetic factors.

**Methods:**

We performed an integrated whole-exome sequencing (WES) analysis of an AT-DILI cohort, combining common variant association analysis, rare variant burden testing at the gene and pathway levels, and exploratory genotype–phenotype analyses. A matched sub-cohort was further analyzed for cis genetic–epigenetic associations between genetic variants and DNA methylation.

**Results:**

Rare variant burden analysis prioritized *PTGS1* as a candidate susceptibility gene (P SKAT-O = 4.58 × 10^-4^, Enrichment Ratio = 3.461), with an increased burden of rare functional variants observed among AT-DILI cases. Pathway-level burden analyses further indicated enrichment of biological processes related to xenobiotic metabolism, immune regulation, and cellular stress responses, including regulated cell death. A cis genetic–epigenetic association analysis in a matched sub-cohort (n = 28) identified a nominal association at the *GPR132* locus, suggesting a potential relationship between genetic variation and DNA methylation. Furthermore, genotype–phenotype analyses showed that rare variant burden in *S100A2* displayed nominal positive correlations with peak alanine aminotransferase (ALT) levels.

**Conclusion:**

These findings provide an integrated characterization of candidate rare variant signals and associated biological processes in AT-DILI. The results prioritize *PTGS1* and other candidate loci for further investigation and expand our understanding of potential genetic contributors to inter-individual variability in anti-tuberculosis treatment outcomes.

## 1 Introduction

Tuberculosis (TB) treatment is frequently complicated by anti-tuberculosis drug–induced liver injury (AT-DILI), the most common serious adverse event limiting therapy. AT-DILI occurs in approximately 2%–28% of treated patients (Kumar et al., 2025; Yang et al., 2026), reflecting substantial heterogeneity across populations and clinical definitions. Although most cases present with asymptomatic elevations in serum transaminases or symptomatic hepatitis, a minority may progress to acute liver failure (ALF), which carries a high mortality rate in the absence of liver transplantation. These risks underscore the need for improved strategies to identify individuals at elevated risk prior to treatment initiation. Marked inter-individual and inter-population variability in AT-DILI susceptibility implicates host genetic factors as key determinants of risk (Prasad et al., 2026). Incidence rates are consistently higher in East Asian populations than in Western cohorts, suggesting population-specific genetic architecture. Among first-line anti-tuberculosis agents, isoniazid (INH) is most frequently associated with severe hepatotoxicity (Lei et al., 2021) and has served as a prototypical compound for pharmacogenomic studies, owing to its well-characterized metabolic activation pathways.

Previous genetic studies of AT-DILI primarily focused on common variants in drug-metabolizing enzymes, highlighting the role of inter-individual differences in xenobiotic biotransformation. Variants in genes such as *NAT2* (Khan et al., 2019) and *CYP2E1* (Bao et al., 2018; Pourmohamadi et al., 2023) have been consistently associated with INH-induced hepatotoxicity, in candidate gene studies, largely through mechanisms involving the accumulation of reactive metabolites. Genome-wide association studies (GWAS) of other drug–induced liver injury (DILI) have successfully identified large-effect HLA variants (e.g., HLA-B*57:01 for flucloxacillin, OR = 36.6) (Yang et al., 2026), implicating adaptive immune responses in disease pathogenesis. However, GWAS of AT-DILI have yielded no such immune signals and have largely replicated known candidate gene associations rather than discovering novel susceptibility genes, partly due to limited sample sizes (e.g., 35–79 cases) (Tarazjani et al., 2025). Consequently, aside from the strong signal at *NAT2*, other identified common variants exhibit modest effect sizes.

Rare functional variants, which often exert larger biological effects than common polymorphisms, may account for a substantial component of the missing heritability not captured by GWAS (Wu et al., 2025). Whole-exome sequencing (WES), coupled with gene-based burden testing, provides an analytical framework to detect the aggregate effects of rare deleterious variants across functionally relevant loci, thereby complementing approaches based on common variant association.

Beyond primary DNA sequence variation, epigenetic modifications have emerged as critical mediators of hepatotoxic responses in AT-DILI. Accumulating evidence suggests that DNA methylation plays an important role in modulating susceptibility to liver injury. In Han Chinese populations, site-specific methylation changes have been associated with AT-DILI risk, while experimental studies have demonstrated that isoniazid exposure induces both global hypomethylation and locus-specific alterations in genes involved in drug metabolism and stress response pathways (Huai et al., 2019; Wei et al., 2020); in animal models, isoniazid exposure induces both genome-wide hypomethylation and locus-specific methylation changes in drug-metabolizing genes (Zhang et al., 2011; Li et al., 2018; de Los Santos-Fernández et al., 2025). Importantly, increasing evidence indicates that genetic variation can shape local DNA methylation through *cis*-regulatory effects, and such genetic-epigenetic interactions have been observed in human hepatocytes at loci involved in drug metabolism (Gotoh-Saito et al., 2025; Singh et al., 2023). However, whether the burden of rare variants at AT-DILI susceptibility loci exerts measurable *cis*-regulatory effects on DNA methylation remains an unexplored dimension of DILI pathogenesis.

In the present study, we conducted a genetic and epigenetic investigation within a well-characterized Han Chinese AT-DILI case-control cohort. Utilizing WES data, we systematically interrogated common variant associations and gene and gene set-based rare variant burden to prioritize candidate genetic signals and biological processes potentially associated with hepatotoxic susceptibility. In a sub-cohort with matched DNA methylation profiling, we further performed an exploratory *cis* genetic–epigenetic association analysis to investigate potential relationships between rare genetic variation and local epigenetic patterns at prioritized loci. These analyses aim to provide a multi-layered characterization of genetic contributions to AT-DILI susceptibility and offer a supportive framework for future genomic risk stratification in anti-tuberculosis therapy.

## 2 Methods

### 2.1 Study design and participants

This study represents an extended investigation based on an AT-DILI cohort that we previously established (Huai et al., 2019). A total of 143 AT-DILI cases from this cohort and 273 control individuals from independent population-based cohorts were subjected to WES using high-quality genomic DNA.

AT-DILI cases were patients receiving standard first-line anti-tuberculosis therapy (2HRZE/4HR regimen: isoniazid, rifampicin, pyrazinamide, and ethambutol for 2 months, followed by isoniazid and rifampicin for 4 months) who developed clinically significant hepatocellular or mixed-pattern liver injury and had a Roussel Uclaf Causality Assessment Method (RUCAM) score ≥6 for inclusion in genetic analyses. Control participants were derived from population-based cohorts previously established by our group (Li et al., 2020), all controls had no documented history of liver disease, viral hepatitis, or prior DILI based on structured medical record review, and were not exposed to anti-tuberculosis drugs.

All participants provided written informed consent. The study protocol was approved by the Ethics Committee of the Bio-X Institutes, Shanghai Jiao Tong University, in accordance with the Declaration of Helsinki.

### 2.2 Whole-exome sequencing, quality control, and variant calling

Genomic DNA was extracted from peripheral blood using standard procedures. WES libraries were constructed using the Agilent SureSelect Human All Exon V8 kit (Agilent Technologies, CA, USA) following capture-based enrichment protocol and sequenced on the Illumina NovaSeq 6000 platform (paired-end 150 bp reads). Raw reads were aligned to the human reference genome (GRCh38/hg38) utilizing the BWA-MEM algorithm (v0.7.17). Subsequent to alignment, PCR duplicates were removed using Picard tools (v2.26). Variant calling was performed jointly on all 143 cases and 273 population-based controls through the GATK HaplotypeCaller (v4.2) pipeline in joint genotyping mode, strictly adhering to the GATK Best Practices. To ensure high-quality variant calls, we employed Variant Quality Score Recalibration (VQSR) supplemented by stringent hard-filtering via VariantFiltration for indels (QualByDepth <2.0) to eliminate potential mapping artifacts and low-confidence calls. Functional annotation was performed via ANNOVAR, integrating allele frequencies from public databases (gnomAD, ExAC, and 1000 Genomes), as well as functional predictions from CADD (v1.6) and REVEL. Genomic evolutionary constraint was assessed using GERP++ scores, where higher values indicate sites under strong purifying selection.

At the sample level, individuals were excluded if the genotype call rate was <98% or if sex inferred from genetic data was inconsistent with clinical records. At the variant level, we retained variants with a call rate ≥95% and a Hardy-Weinberg Equilibrium (HWE) *P*-value >1 × 10^−6^ in the control group.

### 2.3 Population stratification and ancestry assessment

To control for potential confounding from population structure, principal component analysis (PCA) was performed on a pruned subset of high-quality, independent common variants (*r* ^2^ < 0.2). An iterative outlier detection procedure was implemented, defining genetic outliers as samples deviating more than six standard deviations from the population mean across the top ten PCs. To ensure genomic homogeneity, only individuals clustering with the Han Chinese reference population were retained. The first two PCs were subsequently incorporated as covariates in all association models.

### 2.4 Common variant association analysis

Common variants (MAF ≥1%) were analyzed using logistic regression under an additive genetic model implemented in PLINK (v1.9), adjusting for age, sex, and the first two principal components. Genome-wide significance was defined as *P* < 5 × 10^−8^, with a suggestive threshold of *P* < 1 × 10^−5^. The genomic inflation factor (λ_GC_) was calculated to assess residual population stratification.

### 2.5 Gene-based rare variant association analysis

Rare variants were defined as protein-altering variants with a MAF <0.005 in both the study cohort and gnomAD East Asian population. Protein-altering variants included stop-gain, stop-loss, splice-site, frameshift, and deleterious missense variants.

Gene-based association analyses were performed using complementary burden and variance-component methods, including the Combined Multivariate and Collapsing test (CMC), the Price burden test (PRICE), and the Sequence Kernel Association Optimized test (SKAT-O), implemented in KGGSeq (v1.0+). SKAT-O P-values were used as the primary metric for candidate gene prioritization due to their robustness across heterogeneous genetic architectures. All models were adjusted for age, sex, and the top two genomic principal components (PCs 1–2).

To prioritize robust susceptibility signals, candidate genes were required to show nominal significance in SKAT-O (P < 0.05) and to include at least one rare variant carrier among cases. A model convergence score (range: 1–3) was defined as the number of methods (CMC, PRICE, SKAT-O) achieving nominal significance, and genes were ranked accordingly.

An enrichment ratio was calculated to quantify the relative burden of rare variants in cases versus controls:

$$\text{Enrichment Ratio}=\frac{\left(AC_{\text{case}\;}+\varepsilon \right)/N_{\text{case}\;}}{\left(AC_{\text{control}\;}+\varepsilon \right)/N_{\text{control}\;}}$$

where AC denotes alternate allele counts, N denotes total allele counts, and ε=0.5 is a continuity correction. Sensitivity analyses using an expanded threshold (MAF <0.01 ) were performed to assess robustness.

### 2.6 Gene-set based rare variant burden analysis

Gene-set level association analyses were performed to evaluate biological pathways potentially related to with AT-DILI susceptibility using curated gene sets from the Molecular Signatures Database (MSigDB, C2 collection). To minimize potential bias introduced by excessively small or large gene sets, only pathways containing between 3 and 300 genes were retained for analysis, resulting in 7,186 gene sets.

Rare protein-altering variants (minor allele frequency [MAF] < 0.005) were aggregated at the gene-set level. Association testing was performed using the Sequence Kernel Association Test framework implemented in RVtests, applying a beta distribution to weight variants according to allele frequency. A minimum minor allele count (MAC) threshold of three was used to reduce instability arising from extremely rare variants.

Statistical significance was evaluated using permutation testing (10,000 permutations), and empirical P-values (PermP) were used to prioritize pathways. This approach enabled the identification of biologically coherent signals across multiple pathway databases, including KEGG, Reactome, and MGI phenotypic annotations.

### 2.7 Exploratory *cis* genetic–epigenetic association analysis integrating genetic and epigenetic profiles

To investigate the potential relationship between rare genetic variation and DNA methylation, a *cis* genetic–epigenetic association analysis was performed in a sub-cohort of 28 individuals with matched WES and DNA methylation data. Candidate CpG units were derived from prior differential methylation analysis conducted in an independent cohort of AT-DILI cases and controls (n = 50 vs. 50), in which differentially methylated probes were identified based on predefined statistical criteria (as described in our previous study (Huai et al., 2019)). These CpG units were further restricted to genomic regions corresponding to genes exhibiting suggestive signals in gene-based rare variant burden analysis, thereby enabling a hypothesis-driven integration of genetic and epigenetic evidence. For each candidate gene, rare variant carrier status was encoded as a binary variable (0 = non-carrier, 1 = carrier of at least one qualifying variant). Associations between carrier status and methylation levels of CpG sites within a ±500 kb *cis* window were evaluated using Spearman rank correlation. This approach was selected to capture monotonic relationships given the ordinal and discretized nature of methylation measurements derived from the BATMAN algorithm. Empirical *P*-values were obtained through permutation testing (10,000 permutations), and CpG variant pairs with empirical *P* < 0.05 were considered suggestive *cis* genetic–epigenetic associations.

### 2.8 Association between rare variant burden and clinical severity

To evaluate the potential clinical relevance of rare variant signals, Spearman rank correlation analyses were conducted between gene-level rare variant burden and peak biochemical markers of liver injury, including alanine aminotransferase (ALT), aspartate aminotransferase (AST), and alkaline phosphatase (ALP). Rare variant burden was quantified for each gene as the total number of rare protein-altering alleles carried per individual. Given the modest sample size and the exploratory nature of this analysis, associations are presented without formal adjustment for multiple testing. Effect size estimates (Spearman ρ) were provided to characterize the direction and magnitude of potential associations and to inform future validation studies in larger cohorts.

## 3 Results

### 3.1 Study population and clinical characteristics

The study cohort initially comprised 143 patients with AT-DILI and 273 control individuals of Chinese ancestry (Table 1). After sample-level quality control (QC), 139 cases and 269 controls were retained in the following analysis. As shown in Supplementary Figure 1A, cases and controls largely overlapped along the first two principal components, indicating comparable genetic ancestry and no evident population substructure.

### 3.2 Genetic architecture and common variant association analysis

To characterize the contribution of common genetic variation to AT-DILI susceptibility, we tested 119,795 common variants (MAF ≥1%) using logistic regression under an additive model. The quantile-quantile (Q-Q) plot exhibited a genomic inflation factor λ_GC_ of 1.049, indicating that population stratification was well-controlled and the observed statistics followed the expected null distribution (Supplementary Figure 1B). A total of 5 variants reached suggestive significance (*P* < 1 × 10^−6^). Among these, a synonymous variant in *PSPH* (rs75497420, OR = 3.23, 95% CI: 2.084-4.998, *P* = 1.52 × 10^−7^) showed the most stable association with increased ATLI risk (Supplementary Table S1).

We further examined previously reported genetic susceptibility markers associated with AT-DILI in our cohort. For *NAT2*, predicted slow acetylator frequency was 17.3% in cases vs. 15.2% in controls, although none of these associations survived multiple-testing correction, their consistency with established pharmacogenetic mechanisms provides supportive context for the subsequent rare-variant analyses.

### 3.3 Rare variant burden prioritizes candidate susceptibility genes

To investigate the contribution of rare protein-altering variants (MAF <0.005) to AT-DILI susceptibility, gene-based association analyses were performed using SKAT-O, supplemented by CMC and PRICE models. Notably, the top 20 candidate genes ranked by SKAT-O statistics exhibited consistent enrichment of rare damaging variants across all three analytical frameworks (Supplementary Table S2). Among the prioritized genes, *PTGS1* showed the most consistent signal (*P*_*SKAT-O*_ = 4.59 × 10^−4^), with a increased burden of rare variants in cases than controls (13 vs. 7 alternate alleles; enrichment ratio = 3.46). This gene encodes prostaglandin G/H synthase 1 (COX-1), a key enzyme involved in prostaglandin synthesis and inflammatory signaling.

Several genes implicated in immune regulation and inflammatory signaling also showed suggestive rare variant enrichment. *CHUK (IKK-α)*, a central kinase in the NF-κB signaling pathway, exhibited an enrichment ratio of 10.68 (7 vs. 1 alternate alleles; *P*_*SKAT-O*_ = 2.46 × 10^−3^). *NLRC5*, a regulator of MHC class I gene expression and innate immune responses, showed a 4.99-fold increase in variant burden (11 vs. 4 alternate alleles; *P*_*SKAT-O*_ = 4.99 × 10^−3^). Additional candidates included *PIEZO1*, a mechanosensitive ion channel implicated in cellular stress responses, and *SLC2A12*, an insulin-responsive glucose transporter, both of which demonstrated consistent enrichment across models and sensitivity analyses (Figures 1A,B).

Furthermore, to evaluate the population frequency spectra of these prioritized signals, rare variants identified in *PTGS1* and *CHUK* were further annotated against East Asian population reference datasets (1000 Genomes EAS, ExAC EAS, and gnomAD Exome EAS). The majority of the identified variants were ultra-rare or absent in population controls (Supplementary Table S3), supporting the enrichment of rare population-depleted variants underlying these gene-level signals.

### 3.4 Structural annotation of rare variants in prioritized *PTGS1* and *CHUK*

To further characterize the potential biological relevance of prioritized rare variant signals, we examined the structural and domain distribution of rare variants in *PTGS1* and *CHUK*. Rare variants in *PTGS1* were subsequently mapped onto the human COX-1 crystal structure (PDB: 6Y3C) to evaluate the spatial distribution of rare variants (Figure 2A). A total of 12 rare protein-altering variants were identified, including 11 missense substitutions and one frameshift insertion (Table 2). Several variants enriched among AT-DILI cases were located within regions with known structural or functional annotations. Notably, p. G65S was positioned at the interface between the EGF-like and membrane-binding domains (Figure 2B), whereas p. A526S mapped adjacent to the catalytic residue Ser530, which is the critical catalytic and aspirin acetylation residue (Lucido et al., 2016) (Figure 2C). Additional case-enriched variants were distributed throughout the catalytic domain (Figure 2A), suggesting that the burden signal likely reflects the cumulative enrichment of multiple rare variants rather than a single recurrent mutation.

**FIGURE 1:**
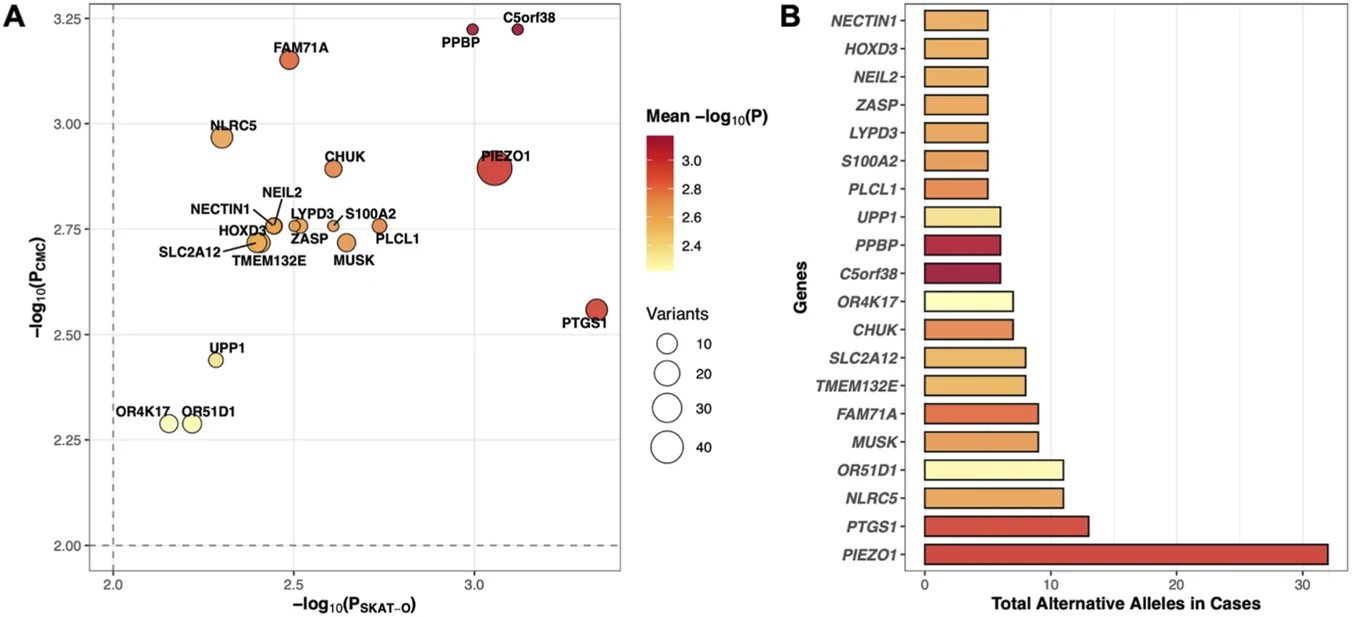
Rare functional variant burden analysis prioritizes candidate AT-DILI candidate susceptibility genes. **(A)** Consistency across gene-based statistical models. Scatter plot comparing gene-level statistical significance -log_10_ (P) values derived from the CMC (y-axis) and SKAT-O (x-axis) models. The top 20 candidate genes were ranked according to *P*_*SKAT-O*_, with concordant signals observed across the three analytical models. Bubble size represents the number of variants identified per gene and the color gradient indicates the mean statistical significance across both models. **(B)** Absolute variant burden in the DILI cohort. Horizontal bar plot displaying the distribution of alternative allele counts strictly within AT-DILI cases for the prioritized top 20 candidate genes.

**FIGURE 2:**
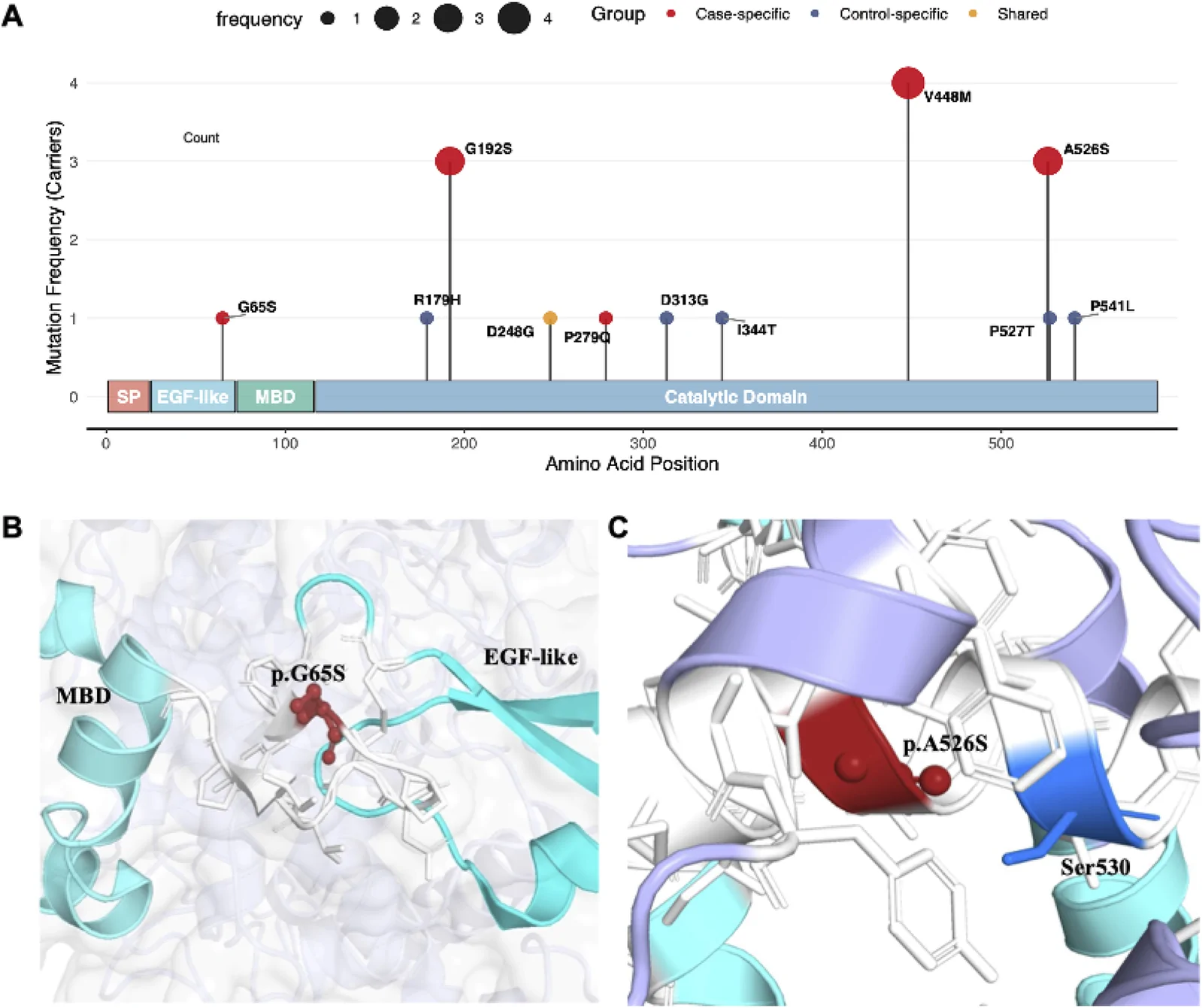
Structural characterization of rare *PTGS1* variants associated with AT-DILI susceptibility. **(A)** Linear schematic representation of the PTGS1 protein. Signal peptide (SP), EGF-like, and membrane-binding domains (MBD) are indicated. The lollipop plot illustrates the distribution of identified rare protein-altering variants; colors represent group specificity (red: case-specific; blue: control-specific; yellow: shared), and sphere heights map to carrier frequencies. **(B)** Detailed conformation of the p. G65S variant. Based on the human PTGS1 crystal structure (PDB: 6Y3C), the p. G65 residue (red sphere) is positioned at the junction of the EGF-like and MBD domains. **(C)** Structural insights into the hydrophobic catalytic center. The yellow sticks represent the native substrate arachidonic acid anchored within the catalytic channel. The p. A526S variant (red sphere) is located adjacent to the critical catalytic residue Ser530 (blue sticks).

**TABLE 1:** Demographic and clinical characteristics of the included patients.

Clinical characteristics	Control (*n* = 269)	Control (%)	Case (*n* = 139)	Case (%)
Age, years^a^	49.67 ± 12.32	-	62.00 ± 6.00	-
Male, *n* (%)	32	11.90	84	60.43
Weight, kg	60.96 ± 10.01	-	49.33 ± 13.05	-
Height, cm	161.69 ± 6.70	-	165.01 ± 7.32	-
Time duration, days	-	-	27.55 ± 6.36	-
Type of DILI	​	-	​	-
Hepatocellular	-	-	34	24.46
Cholestatic	-	-	90	64.75
Mixed	-	-	15	10.79
Liver enzyme levels in hepatocellular DILI cases	​	-	​	-
ALT (IU/L)^b^	-	-	221 ± 42.81	-
ALP (IU/L)	-	-	86 ± 12.60	-
Liver enzyme levels in cholestatic DILI cases	​	-	​	-
ALT (IU/L)^b^	-	-	33 ± 5.48	-
ALP (IU/L)	-	-	226 ± 11.48	-

ALT, alanine aminotransferase; ALP, alkaline phosphatase; ULN, upper limit of normal. The upper limits of normal (ULNs) were defined according to the clinical laboratory of our institution reference ranges (ALT, 40 U/L and ALP, 120 U/L).

^a^ Time duration, drug administration duration of participants before blood collection.

^b^ Data were shown as mean ± SEM., The pattern of DILI, is defined by R ratio ([(ALT/ULN_ALT)/(ALP/ULN_ALP)]). A hepatocellular pattern is defined by R ≥ 5 or ALT >2 ULN; a cholestatic pattern is defined by R ≤ 2; and a mixed pattern is defined by 2 < R < 5.

**TABLE 2:** Functional prediction and conservation analysis of variants in *PTGS1* and *CHUK* genes.

Gene	cDNA change	Protein change	Variant type	REVEL	CADD	GERP++
*PTGS1*	c.193G>A	p.G65S	Missense	0.73	**32.00**	5.70
​	c.536G>A	p.R179H	Missense	0.34	**20.60**	−0.23
​	c.574G>A	p.G192S	Missense	0.46	**24.90**	5.16
​	c.743A>G	p.D248G	Missense	0.50	**26.10**	4.82
​	c.836C>A	p.P279Q	Missense	0.47	**20.10**	4.07
​	c.T873ins + G	p.F291fs	Frameshift	​	​	​
​	c.938A>G	p.D313G	Missense	0.77	**28.50**	5.30
​	c.1031T>C	p.I344T	Missense	0.63	**24.70**	5.58
​	c.1342G>A	p.V448M	Missense	0.14	18.88	0.40
​	c.1576G>T	p.A526S	Missense	0.36	**23.60**	5.41
​	c.1579C>A	p.P527T	Missense	0.46	**25.30**	5.41
​	c.1622C>T	p.P541L	Missense	0.59	**28.60**	5.41
*CHUK*	c.1496C>T	p.T499M	Missense	0.17	**22.90**	5.70
	c.1059A>C	p.E353D	Missense	0.12	14.43	−1.39
	c.622G>A	p.G208R	Missense	0.81	**26.80**	5.07
	c.442G>A	p.E148K	Missense	0.47	**31.00**	5.85
	c.59G>A	p.R20Q	Missense	0.21	**26.40**	3.69
	c.32C>T	p.A11V	Missense	0.10	**23.10**	3.79

REVEL, rare exome variant ensemble learner; CADD, combined annotation-dependent depletion; GERP++, genomic evolutionary rate profiling; “.”, not applicable for frameshift insertions. Bold values indicate predicted deleterious effects based on a CADD score threshold of > 20.

Given the enrichment of immune-regulatory pathways in the burden analysis, we next examined the distribution of rare variants across functional domains of *CHUK* (Supplementary Figure S1). Several variants clustered within the N-terminal kinase domain of IKK-α, a region essential for NF-κB pathway activation. Among these, p. G208R and p. E148K, localized within the N-terminal kinase domain and affected evolutionarily conserved residues (GERP++ > 5.0) with high predicted deleteriousness scores (REVEL = 0.812 and 0.471; CADD = 26.8 and 31.0, respectively). In addition, p. R20Q localized within the same functional region, further supporting a notable spatial clustering of case-specific rare variants within the critical kinase domain. These observations raise the possibility that rare coding variation in *CHUK* may influence protein structure or regulatory functions.

Overall, these structural and domain-centric analyses support the functional relevance of prioritized rare variants signals in *PTGS1* and *CHUK* by demonstrating their localization within evolutionarily conserved or biologically important regions, although experimental validation is required.

### 3.5 Network analysis characterization of rare variant-associated genes and DILI-Related functional modules

To explore the functional relationships among genes prioritized from rare variant burden analysis, we constructed a protein–protein interaction (PPI) network using STRING v12.0, integrating prioritized burden genes with well-established DILI-related genes, including drug-metabolizing enzymes, transporters, and immune regulators (Figure 3A).

**FIGURE 3:**
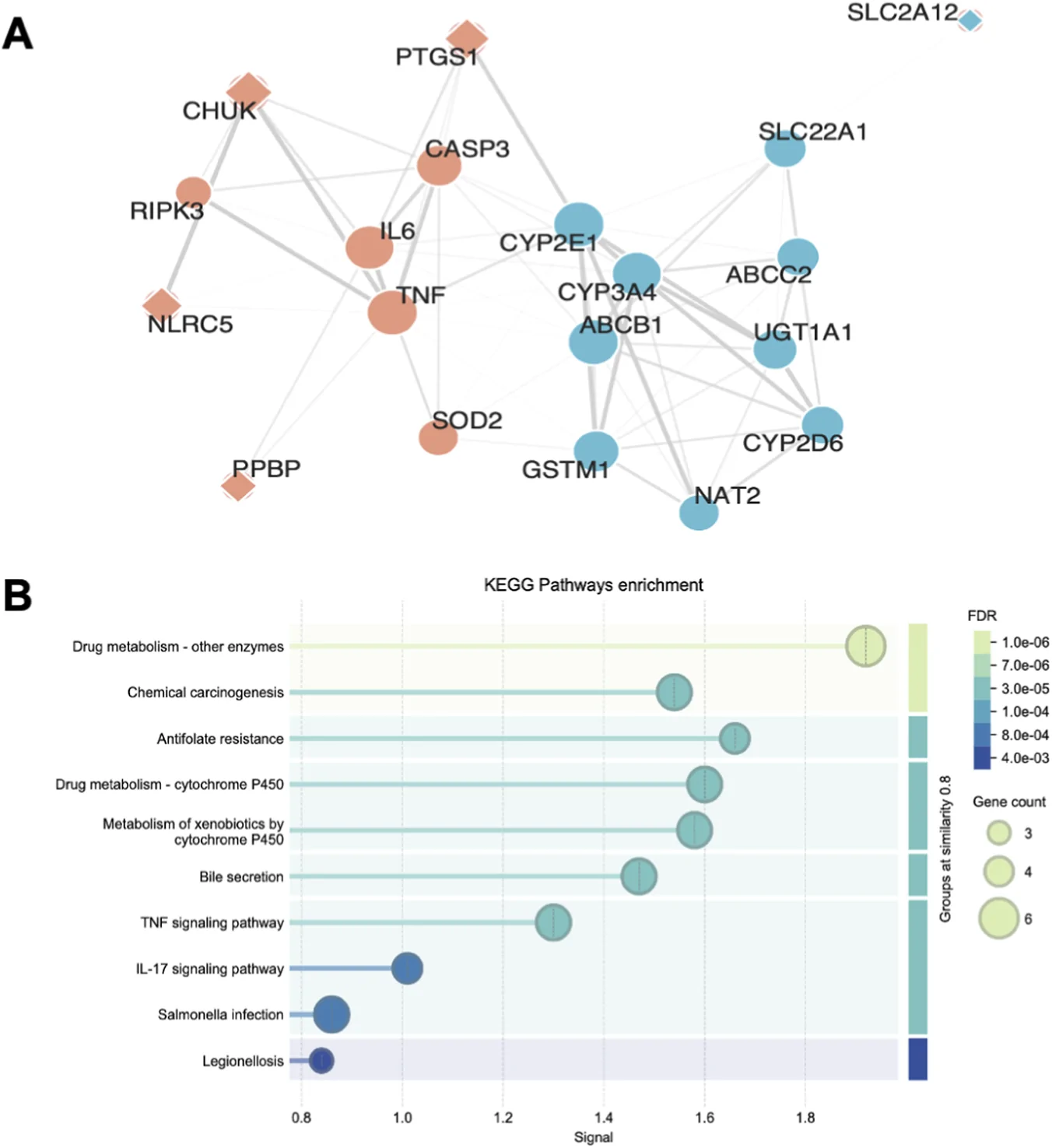
Protein-protein interaction (PPI) network and functional annotation of rare variant-associated genes. **(A)** PPI network landscape. Red and blue circles represent two distinct functional modules identified by the Louvain community detection algorithm. Diamonds highlight genes enriched with rare variant burdens in the current cohort (*PTGS1, PPBP, CHUK, SLC2A12*, and *NLRC5*). Node size is proportional to the degree of connectivity. **(B)** KEGG pathway enrichment analysis. The lollipop plot illustrates representative enriched pathways for the network components. The X-axis represents the enrichment signal, and the color gradient indicates the False Discovery Rate (FDR). Bubble size reflects the number of genes involved in each pathway. Modules are grouped at a similarity threshold of 0.8.

KEGG pathway enrichment analysis was subsequently performed to characterize the biological pathways and processes represented within the network (Figure 3B). The primary module was predominantly enriched for pathways related to xenobiotic metabolism and hepatic detoxification, including *Drug metabolism–cytochrome P450*, *Metabolism of xenobiotics by cytochrome P450*, and *Bile secretion*. This module comprised canonical phase I/II metabolic enzymes and transport-associated genes, including *CYP2E1, CYP3A4, NAT2, GSTM1, ABCB1,* and *ABCC2*. Additionally, the secondary module demonstrated enrichment for inflammatory and immune-regulatory pathways, specifically *TNF signaling* and *IL-17 signaling*, reflecting generalized cellular stress and inflammatory response pathways.

To further describe the topological properties of individual nodes, we performed quantitative network hub centrality analysis using degree and betweenness centrality metrics (Supplementary Table S4). Established DILI-associated genes, including *CYP2E1, TNF,* and *ABCB1*, exhibited the highest degree and betweenness centrality values, consistent with their broad involvement in hepatic metabolism and inflammatory regulation. Among the rare-variant burden genes, *CHUK* displayed relatively elevated betweenness centrality despite a moderate degree profile, positioning it near the topological interface of inflammatory and stress-response subnetworks. Given the established role of *CHUK* (IKK-α) in NF-κB signaling, this network position suggests a potential intersection with inflammatory stress pathways.

In contrast, several rare variant-associated genes, including *PTGS1*, *NLRC5*, and *PPBP*, occupied relatively peripheral network positions, reflecting lower global betweenness centrality. Nevertheless, *PTGS1* resided within a functional interaction neighborhood containing metabolic enzymes, inflammatory mediators, and apoptotic effectors, including *CYP2E1*, *TNF*, *IL6*, and *CASP3*. This localized connectivity pattern is consistent with a potential role for prostaglandin signaling in orchestrating inflammatory and cell death responses following hepatocellular stress (Chen et al., 2022). Although these topological relationships do not imply direct causal regulation, they provide a supportive systems-level context for the structural and burden-based findings observed in the present study.

Together, this network architecture suggests that rare variant-burden genes concentrate within interconnected pathways governing xenobiotic metabolism, hepatic transport, inflammatory signaling, and hepatocellular stress. Rather than acting in isolation, these topological patterns suggest potential functional crosstalk across metabolic/transport processes and immune-regulatory pathways, providing a systems-level perspective on inter-individual susceptibility to AT-DILI.

### 3.6 Pathway-level burden analysis highlights rare variant-enriched biological processes in AT-DILI

To move beyond single-gene associations and explore coordinated biological modules underlying AT-DILI, we performed a gene-set-based rare variant association analysis utilizing 7,186 curated pathways from the Molecular Signatures Database (MSigDB). Rare functional variants were aggregated at the pathway level and evaluated via a permutation-based burden test.

This analysis highlighted the top 20 candidate pathways, illustrating a structured organization of genetic susceptibility at the systems level. These pathways could be broadly grouped into four functional categories: *Disease Association*, *Molecular Mechanism*, *Cellular Process*, and *Other Regulatory Programs* (Figure 4). Specifically, within the *Disease Association* and *Other Regulatory Programs* domains, we observed significant genetic enrichment in drug and lipid metabolism, including *acetaminophen Metabolism* and *Linoleic Acid Metabolism*. These enriched pathways aligns closely with recent human toxicogenomics evidence suggesting that the dysregulation of lipid, amino acid, and tryptophan metabolic networks represents characteristic cellular features associated with DILI (Bergen et al., 2025).

**FIGURE 4:**
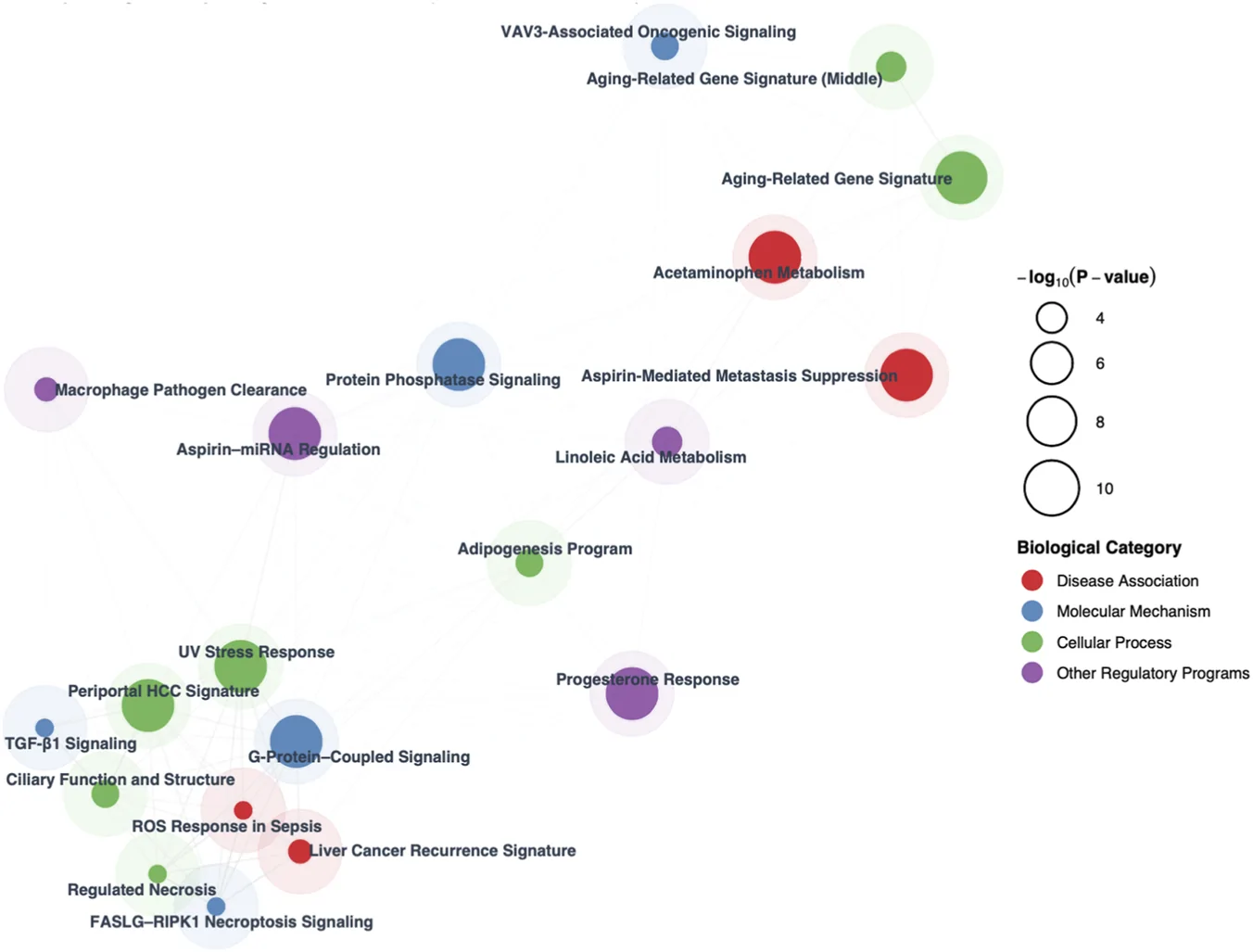
Hierarchical Crosstalk Network of Genetic Burden Pathways. Integrated analysis of shared genetic burden identifies a multi-dimensional landscape of risk pathways. Nodes represent top 20 significant pathways identified by burden analysis, and edges represent the similarity between pathways, calculated as the Jaccard similarity coefficient based on shared gene membership. Node color: Denoting biological categories (red: disease-associated pathways; blue: molecular mechanisms; green: cellular processes; purple: other regulatory programs). The background shading highlights the functional clustering of these domains. Node Size: Proportional to the enrichment significance, represented as -log_10_(P-value). Edge Transparency: Represents the strength of crosstalk (Jaccard index); denser lines indicate a higher degree of shared genetic architecture between pathways.

Several enriched *Cellular Process* pathways were related to cellular stress response and DNA damage, such as *ROS Response in Sepsis* and *UV Stress Response*, which involve homeostasis-maintaining genes (e.g., *NEIL2* and *CHUK*). Furthermore, pathways regulating cell death and tissue response, particularly *Regulated Necrosis* and *FASLG–RIPK1 Necroptosis Signaling*, were also enriched. These findings suggest that rare variant burden may be associated with cellular injury responses; however, the contribution of specific cell death pathways requires further functional investigation.

Immune-related pathways were also enriched, including *TGF-β1 signaling* and *Macrophage Pathogen Clearance,* suggesting potential involvement of immune regulatory processes in modulating hepatic injury responses. Additionally, several enriched gene sets were derived from liver-related disease and transcriptional signatures, such as the *Periportal HCC Signature* and *Ciliary-associated programs* (Supplementary Table S5). These likely reflect shared downstream biological states associated with hepatic stress and tissue remodeling. Overall, these results suggest that rare variant burden-associated signals are distributed across multiple biological processes involving metabolism, stress response, immune regulation and cell death, providing a systems-level framework that complements the gene-based findings.

### 3.7 Exploratory *cis* genetic–epigenetic association suggests a potential relationship at the *GPR132* locus

An exploratory *cis* genetic–epigenetic association signal was observed at the *GPR132* locus (chr14:105051371; CADD = 23.1) using matched WES and DNA methylation profiles from a sub-cohort (n = 28). Within a ±500 kb *cis*-window, rare variant carrier status exhibited associations with multiple proximal CpG units (Figure 5A). Specifically, carrier status showed a positive correlation with probe A_17_P09529823 (Spearman ρ = 0.4589, *P* = 0.0377) and negative correlations with A_17_P16739872 (ρ = −0.5007, *P* = 0.0379), A_17_P16740256 (ρ = −0.480, *P* = 0.0547). Stratified analysis further demonstrated differences in methylation state distributions between non-carriers and carriers across these three leading CpG sites (Figure 5B). Although these associations did not withstand multiple testing correction, the directional consistency across several proximal CpG sites raises the possibility of a localized relationship between genetic variation and DNA methylation at this locus.

**FIGURE 5:**
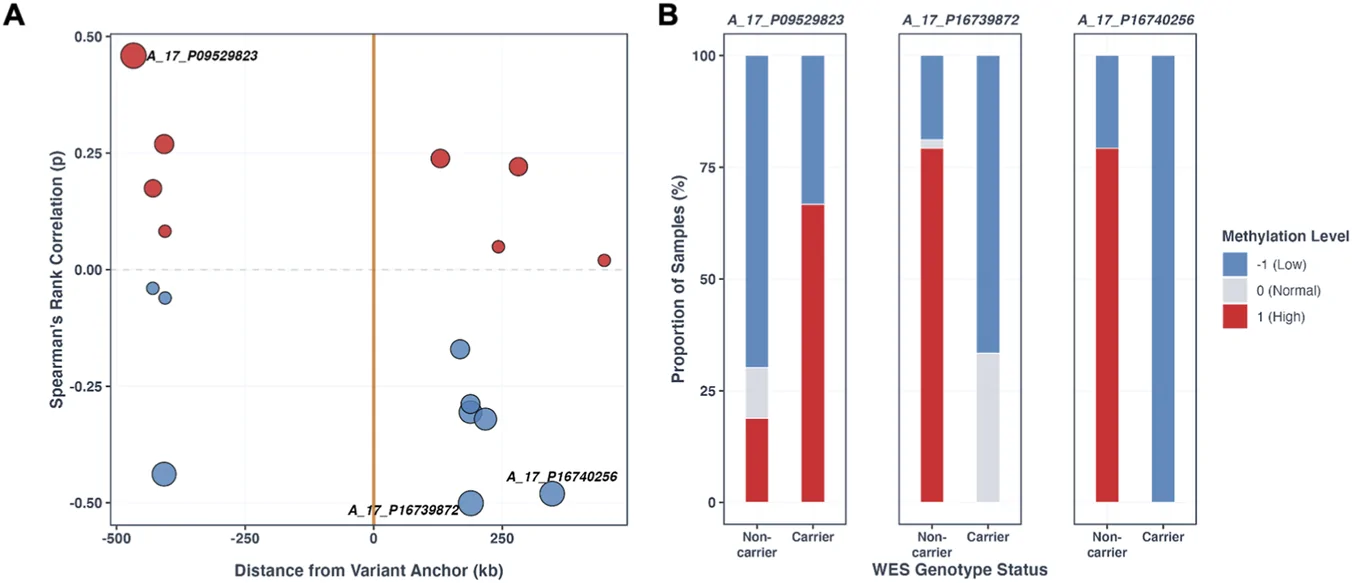
Exploratory *cis* Genetic–Epigenetic Association Analysis at the *GPR132* Locus. **(A)** Regional association plot of the *GPR132* locus. The y-axis shows the Spearman correlation coefficient (ρ) between variant carrier status and proximal CpG methylation states within a ±500 kb *cis*-window. Bubble size represents the magnitude of the permutation-based p-value (-log_10_ (*P*_*perm*_)). Markers are color-coded by the direction of association (red: positive correlation; blue: negative correlation). **(B)** Relative distribution of DNA methylation levels stratified by WES genotype status. Bar plots illustrate the proportion of samples classified with low (blue), normal (grey), or high (red) methylation levels for the three leading probes (A_17_P09529823, A_17_P16739872, and A_17_P16740256) among non-carriers and carriers.

### 3.8 Association between rare variant burden and clinical severity

To evaluate the potential clinical relevance of the rare variant burden signals, we performed correlation analyses between gene-level variant burden and peak biochemical markers of liver injury (ALT, AST and ALP). Among the candidate loci, the rare variant burden in *S100A2* showed a modest positive correlation with peak ALT levels (Spearman ρ = 0.23, *P =* 0.0041) (Figure 6A). This association was further supported by a carrier-level comparison, where rare variant carriers exhibited higher clinical liver injury severity compared with non-carriers (*P* = 0.0198, Figure 6B). *S100A2* encodes a calcium-binding protein reported to act as a Damage-Associated Molecular Pattern (DAMP), that promotes inflammatory cell recruitment and secondary hepatic inflammation following tissue stress. These correlations, while exploratory, suggest that rare variants at specific loci may influence not only the initial susceptibility to AT-DILI but also the magnitude of subsequent hepatocellular damage, contributing to the observed phenotypic heterogeneity in clinical settings.

**FIGURE 6:**
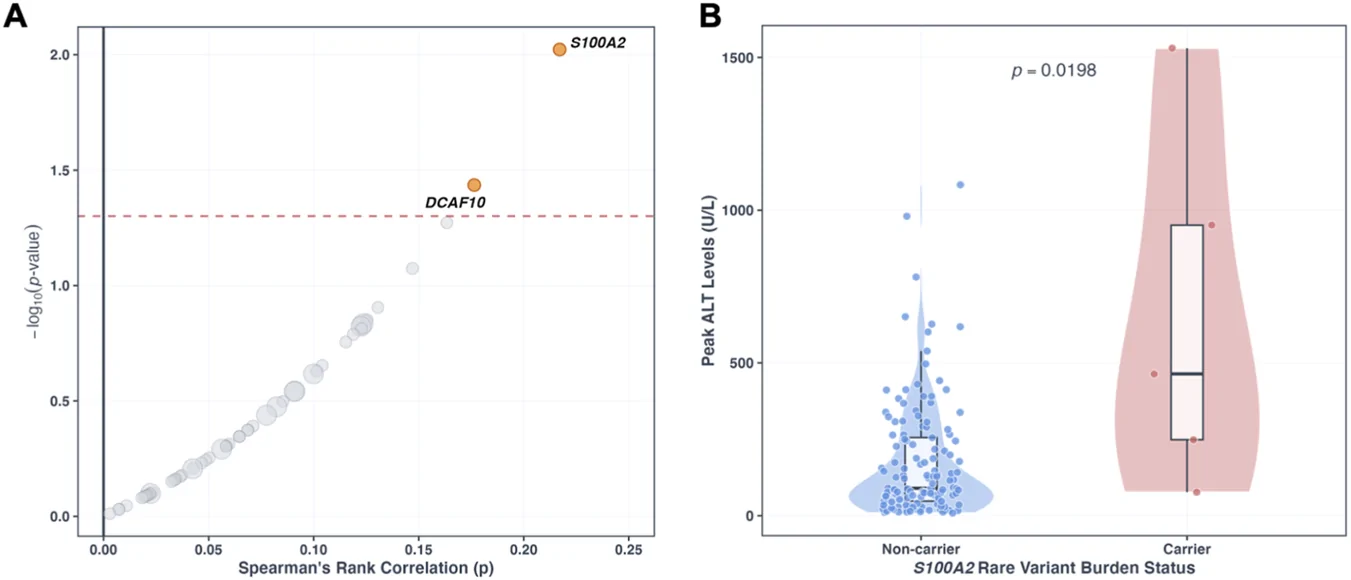
Association between rare variant burden and biochemical severity markers in AT-DILI. **(A)** Association landscape between gene-level rare variant burden and clinical phenotypes. The x-axis represents Spearman’s rank correlation coefficient (ρ), and the y-axis denotes −log_10_(*P*-value). The red dashed line indicates the nominal significance threshold (*P* < 0.05). **(B)** Comparison of peak ALT levels (U/L) between non-carriers and carriers of rare variants in *S100A2*. Violin plots depict the distribution of ALT levels, with embedded boxplots indicating median and interquartile range (IQR); whiskers extend to 1.5× IQR. Individual data points are shown as dots.

## 4 Discussion

DILI remains a major limitation in anti-tuberculosis therapy, with substantial inter-individual variability in susceptibility that is not fully explained by known genetic factors. In the present study, we performed an integrated analysis combining common variant association, rare variant burden testing, pathway-level aggregation, and exploratory epigenetic interrogation in a well-characterized AT-DILI cohort. Our findings provide a systems-level characterization of candidate susceptibility signals, suggesting that the rare functional variants may converge on metabolic, immune, and cellular stress response pathways.

Rare variant burden testing prioritized *PTGS1* (encoding COX-1) as the top-ranked candidate susceptibility gene. COX-1 derived prostaglandin E_2_ (PGE_2_) has been shown to exert hepatoprotective effects by supporting mitochondrial integrity, attenuating pro-apoptotic signaling, and modulating macrophage-mediated inflammatory responses (Xiao et al., 2015). In murine models, genetic or pharmacological disruption of COX-1 exacerbates chemically induced liver injury, whereas exogenous PGE_2_ partially rescues the phenotype (Xiao et al., 2015). The enrichment of rare missense and loss-of-function variants in *PTGS1* among AT-DILI cases raises the possibility that altered prostaglandin-mediated cytoprotective responses may contribute to susceptibility to drug-induced hepatic stress (Lucena et al., 2019). However, in the absence of direct functional validation, this association should be interpreted as a prioritized genetic susceptibility signal rather than evidence of a causal mechanism.

At the pathway level, rare variant burden analyses further identified enrichment of biological processes related to xenobiotic metabolism, lipid metabolism, and inflammatory regulation. These findings provide additional biological context for interpreting the rare variant burden signals but do not establish direct biochemical interactions between *PTGS1* and classical drug-metabolizing enzymes.

Beyond metabolic processes, rare variant enrichment was also observed in genes involved in immune signaling, including *CHUK* and *NLRC5*, implicating inflammatory pathways and antigen presentation processes. At the pathway level, enrichment of *Macrophage Pathogen Clearance* and *ROS Response* further supports a role for innate immune activation in disease susceptibility. These findings suggest that rare genetic variation may influence processes related to hepatocyte stress, immune activation, and tissue repair processes during drug-induced injury.

The co-enrichment of *Regulated Necrosis* and *FASLG-RIPK1 Necroptosis Signaling* pathways reflects known cell death cascades activated during severe hepatocellular stress (Kondo et al., 2021). Unlike apoptosis, necroptosis releases DAMPs that amplify Kupffer cell activation and neutrophil recruitment, establishing a feed-forward inflammatory loop that may explain disproportionate injury severity in genetically susceptible individuals. In this context, the exploratory association between *S100A2* rare variant burden and peak ALT levels further suggests a possible relationship between rare genetic variation and inter-individual differences in biochemical injury severity. These observations should be interpreted cautiously but may provide a basis for further investigation of genotype–phenotype relationships in AT-DILI.

The *cis* genetic–epigenetic association at the *GPR132* locus provides preliminary evidence of a possible relationship between rare genetic variation and local DNA methylation patterns. Although the associations did not survive multiple testing correction, the consistent methylation changes across several proximal CpG sites hint at a localized epigenetic trend. GPR132 is implicated in macrophage activation and inflammatory signaling. Recent experimental studies have shown that *Gpr132* ablation attenuates hepatic inflammation and parenchymal injury in DILI models (Cheng et al., 2026), whereas its perturbation in preclinical systems disrupts biliary lipid homeostasis and CYP7A1-mediated bile acid synthesis (Johnson et al., 2008). Given the reported roles of *GPR132*, this locus represents a biologically plausible candidate for future functional investigation.

Despite the integrative multi-omics framework and convergent genetic, epigenetic and clinical signals identified in this study, several limitations should be acknowledged. First, the relatively modest sample size imposes constraints on statistical power, particularly for rare variant and epigenetic analysis. Accordingly, the current findings should be interpreted cautiously and require independent replication in larger and better-balanced cohorts. Second, the use of peripheral blood for methylation profiling may not fully capture liver-specific regulatory processes. Third, functional validation was not performed, and the biological mechanisms inferred from genetic associations remain to be experimentally confirmed. Fourth, detailed longitudinal information on concomitant medication exposure, particularly NSAIDs targeting the COX-1 pathway, was unavailable for all participants, and residual medication-related confounding of the *PTGS1* association cannot be completely excluded. Finally, the current study cohort was exclusively restricted to Han Chinese individuals. Due to the complex and divergent genetic architecture of drug metabolism and immune responses across different ancestries, the generalizability of these candidate markers across diverse ancestral populations remains uncertain. Future studies integrating larger cohorts and functional validation, as well as liver-relevant omics data including regulatory, proteomic, and metabolomic layers, will be important to further clarify the biological basis of AT-DILI susceptibility.

In summary, this study provides an integrated assessment of genetic susceptibility to AT-DILI. By integrating multiple layers of genetic, pathway-level, and epigenetic profiling, our findings provide a supportive systems-level framework for understanding inter-individual variability in drug-induced liver injury. These insights may ultimately contribute to improved risk stratification and safer therapeutic strategies in tuberculosis treatment.

## Data Availability

The original contributions presented in the study are publicly available. This data can be found here: National Genomics Data Center (NGDC), China National Center for Bioinformation, under accession number HRA021323.
