# Supplementary material for: Whole-exome sequencing reveals cumulative rare variant burden associated with susceptibility to anti-tuberculosis drug-induced liver injury in a Han Chinese cohort
Source: Front Pharmacol. 2026 Oct 12;17:1952049. doi: 10.3389/fphar.2026.1952049 (PMC13720600; doi:10.3389/fphar.2026.1952049)
Supplement: Supplementary file 1 [file Supplementaryfile1.docx]

Supplementary Material

**Supplementary Figure 1. Population Stratification and Common Variant Association Results for AT-DILI.** **(A)** Principal Component Analysis (PCA) Plot. Cases (red) and controls (blue) largely overlap along the first two principal components, indicating comparable genetic ancestry and successful control of population stratification. **(B)** Quantile-Quantile (Q-Q) Plot of Common Variants. **(C)** Manhattan Plot of Common Variant Association. The y-axis shows the -log_10_ (*P*) for individual SNPs, arranged by chromosomal position on the x-axis. The solid red line indicates the genome-wide significance threshold (*P* < 5 × 10⁻^8^), and the solid blue line indicates the suggestive significance threshold (*P* < 1 × 10^⁻5^).

**Table S1. Top significant SNPs and their functional annotations.**

| **SNP** | **Gene** | **cDNA** | **ExonicFunction** | ***P*** | **OR (95% CI)** |
| --- | --- | --- | --- | --- | --- |
| chr19: 4511528 | *PLIN4* | c.2235_2432del | nonframeshift deletion | 4.793 × 10⁻^10^ | 4.266 (2.636-6.903) |
| chr10: 117167688 | *MIR3663* | . | . | 1.609× 10⁻^9^ | 0.103 (0.049-0.215) |
| chr7: 56021087 | *PSPH* | c.C126T | synonymous SNV | 1.518 × 10⁻^7^ | 3.225 (2.038-5.102) |

**Table S2. Gene-Based Association Results for Rare Functional Variants (MAF < 0.005) in the AT-DILI Discovery Cohort.**

| **Gene** | **SKAT-O *P*** | **CMC *P*** | **Price *P*** | **Enrichment Ratio** | **Variant Consequence** |
| --- | --- | --- | --- | --- | --- |
| *PTGS1* | 4.589 × 10⁻^4^ | 2.763 × 10⁻^3^ | 5.400 × 10⁻^3^ | 3.461 | Missense; Frameshift |
| *C5orf38* | 7.589 × 10⁻^4^ | 5.970 × 10⁻^3^ | 8.50 × 10⁻^3^ | 43.165 | Missense |
| *PIEZO1* | 8.790 × 10⁻^4^ | 1.275 × 10⁻^3^ | NA | 2.451 | Missense |
| *PPBP* | 1.012 × 10⁻^3^ | 5.970 × 10⁻^3^ | 6.500 × 10⁻^4^ | 43.165 | Missense |
| *PLCL1* | 1.831 × 10⁻^3^ | 1.749 × 10⁻^3^ | 2.500 × 10⁻^3^ | 35.971 | Missense |
| *MUSK* | 2.260 × 10⁻^3^ | 1.916 × 10⁻^3^ | 2.600 × 10⁻^3^ | 7.676 | Missense; Splicing |
| *CHUK* | 2.456 × 10⁻^3^ | 1.280 × 10⁻^3^ | 1.200 × 10⁻^3^ | 10.675 | Missense |
| *S100A2* | 2.459 × 10⁻^3^ | 1.749 × 10⁻^3^ | 1.700 × 10⁻^3^ | 35.971 | Missense |
| *LYPD3* | 3.033 × 10⁻^3^ | 1.749 × 10⁻^3^ | 2.600 × 10⁻^3^ | 35.971 | Missense |
| *ZASP* | 3.146 × 10⁻^3^ | 1.749 × 10⁻^3^ | 2.000 × 10⁻^3^ | 35.971 | Missense |
| *FAM71A* | 3.253 × 10⁻^3^ | 7.050 × 10⁻^3^ | 9.000 × 10⁻^4^ | 7.676 | Missense |
| *NEIL2* | 3.585 × 10⁻^3^ | 1.749 × 10⁻^3^ | 1.950 × 10⁻^3^ | 35.971 | Missense |
| *HOXD3* | 3.585 × 10⁻^3^ | 1.749 × 10⁻^3^ | 2.500 × 10⁻^3^ | 35.971 | Missense |
| *NECTIN1* | 3.591 × 10⁻^3^ | 1.749 × 10⁻^3^ | 2.200 × 10⁻^3^ | 35.971 | Missense |
| *TMEM132E* | 3.914 × 10⁻^3^ | 1.916 × 10⁻^3^ | 4.150 × 10⁻^3^ | 6.823 | Missense |
| *SLC2A12* | 4.000 × 10⁻^3^ | 1.916 × 10⁻^3^ | 4.050 × 10⁻^3^ | 6.823 | Missense |
| *NLRC5* | 4.997 × 10⁻^3^ | 1.078 × 10⁻^3^ | NA | 4.987 | Missense |
| *UPP1* | 5.191 × 10⁻^3^ | 3.636 × 10⁻^3^ | 3.000 × 10⁻^3^ | 9.150 | Missense |
| *OR51D1* | 6.041 × 10⁻^3^ | 5.145 × 10⁻^3^ | 1.800 × 10⁻^3^ | 9.382 | Missense |
| *OR4K17* | 7.004 × 10⁻^3^ | 5.145 × 10⁻^3^ | 2.400 × 10⁻^3^ | 9.570 | Missense |

**# CMC**: Combined Multivariate and Collapsing test; **PRICE**: Probability-Ratio Information Criterion for Enrichment; **SKAT-O**: Optimal Sequence Kernel Association Test. **Enrichment Ratio**: Calculated as the relative burden of rare variants in cases versus controls. **MAF**: Minor Allele Frequency; **NA**: Not available due to computational convergence limitations.

**Table S3. External population allele frequencies of candidate rare functional variants underlying gene-level burden signals in *PTGS1* and *CHUK.***

| **Gene** | **cDNA Change** | **Protein Change** | **1000G EAS AF** | **ExAC EAS AF** | **gnomAD EAS AF** |
| --- | --- | --- | --- | --- | --- |
| *PTGS1* | c.193G>A | p.G65S | N | 0.000 | 0.000 |
|  | c.536G>A | p.R179H | N | 0.000 | 0.000 |
|  | c.574G>A | p.G192S | 0.002 | 0.002 | 0.003 |
|  | c.743A>G | p.D248G | 0.001 | 0.003 | 0.003 |
|  | c.836C>A | p.P279Q | N | N | N |
|  | c.T873ins+G | p.F291fs | N | N | N |
|  | c.938A>G | p.D313G | N | 0.000 | 0.000 |
|  | c.1031T>C | p.I344T | N | 0.000 | N |
|  | c.1342G>A | p.V448M | 0.003 | 0.003 | 0.003 |
|  | c.1576G>T | p.A526S | 0.002 | 0.003 | 0.004 |
|  | c.1579C>A | p.P527T | N | 0.000 | 0.000 |
|  | c.1622C>T | p.P541L | N | 0.000 | 0.000 |
| *CHUK* | c.1496C>T | p.T499M | N | 0.000 | 0.000 |
|  | c.1059A>C | p.E353D | 0.003 | 0.004 | 0.005 |
|  | c.622G>A | p.G208R | N | N | N |
|  | c.442G>A | p.E148K | N | N | N |
|  | c.59G>A | p.R20Q | N | N | N |
|  | c.32C>T | p.A11V | N | 0.005 | 0.004 |

**#External Reference Populations:** Minor allele frequencies (MAF) in East Asian populations were extracted from 1000 Genomes (1000G EAS, 2013-05 release), Exome Aggregation Consortium (ExAC EAS), and Genome Aggregation Database Exome (gnomAD exome EAS v2.1.1). **N:** Not Recorded (indicates novel variants or variants not captured in the specified reference population databases).

**Supplementary Figure 2. Schematic domain structure and rare variant distribution of CHUK.** Linear programmatic model of the CHUK protein (IKK-α, 745 amino acids), illustrating the N-terminal Kinase Domain (blue, residues 15-302), Leucine Zipper (LZ, green, residues 455-476), Helix-Loop-Helix domain (HLH, light green, residues 603–652), and the NEMO-Binding Domain (NBD, orange, residues 738-743). Lollipop structures map the precise locations of the 6 identified rare variants from our cohort (Table 2). Stem heights reflect the relative magnitudes of predicted deleteriousness (CADD scores). Coral-colored spheres denote case-specific/enriched functional variants (e.g., *p.E148K* and *p.G208R* clustering tightly inside the essential kinase catalytic framework), whereas blue spheres indicate variants observed exclusively in control individuals.

**Table S4. Network topological properties of genes in the PPI network.**

| **Gene Symbol** | **Degree Centrality** | **Betweenness Centrality** | **Degree Percentile Rank** | **Betweenness Percentile Rank** |
| --- | --- | --- | --- | --- |
| *CYP2E1* | 12 | 0.268 | 0.889 | 1.000 |
| *TNF* | 12 | 0.229 | 0.889 | 0.944 |
| *CYP3A4* | 11 | 0.137 | 0.778 | 0.889 |
| ***CHUK*** | 5 | 0.111 | 0.278 | 0.778 |
| *SLC22A1* | 7 | 0.111 | 0.444 | 0.778 |
| *IL6* | 11 | 0.065 | 0.778 | 0.722 |
| *CASP3* | 9 | 0.059 | 0.667 | 0.667 |
| *ABCB1* | 12 | 0.033 | 0.889 | 0.611 |
| *GSTM1* | 9 | 0.020 | 0.667 | 0.556 |
| *CYP2D6* | 7 | 0.013 | 0.444 | 0.500 |
| *ABCC2* | 7 | 0.000 | 0.444 | 0.000 |
| *NAT2* | 6 | 0.000 | 0.333 | 0.000 |
| ***NLRC5*** | 3 | 0.000 | 0.111 | 0.000 |
| ***PPBP*** | 2 | 0.000 | 0.056 | 0.000 |
| ***PTGS1*** | 4 | 0.000 | 0.167 | 0.000 |
| *RIPK3* | 4 | 0.000 | 0.167 | 0.000 |
| *SOD2* | 6 | 0.000 | 0.333 | 0.000 |
| *UGT1A1* | 8 | 0.000 | 0.611 | 0.000 |
| ***SLC2A12*** | 1 | 0.000 | 0.000 | 0.000 |

*Note*: Genes in bold and italics indicate genes that were statistically significant in the rare variant burden analysis (as detailed in Table S2).

**Table S5. Detailed results of pathway level burden analysis.**

| **Pathway** | ***P*-value** | ***PermP*-value** |
| --- | --- | --- |
| Acetaminophen Metabolism | 2.68 × 10⁻⁴ | < 0.0001 |
| Protein Phosphatase Signaling (SPPA) | 3.49 × 10⁻⁴ | < 0.0001 |
| Periportal HCC Signature | 2.49 × 10⁻^3^ | < 0.0001 |
| Aging Related Gene Signature | 5.94 × 10⁻⁴ | < 0.0001 |
| UV Stress Response | 1.83 × 10⁻^3^ | < 0.0001 |
| Aspirin miRNA Regulation | 5.49 × 10⁻⁴ | < 0.0001 |
| Aspirin-Mediated Metastasis Suppression | 2.19 × 10⁻⁴ | < 0.0001 |
| G-Protein Coupled Signaling | 1.46 × 10⁻^3^ | < 0.0001 |
| Progesterone Response (Temporal) | 5.07 × 10⁻⁴ | < 0.0001 |
| Adipogenesis Program | 1.38 × 10⁻^3^ | 1.00× 10⁻⁴ |
| VAV3 Associated Oncogenic Signaling | 5.93 × 10⁻⁴ | 1.00× 10⁻⁴ |
| Ciliary Function and Structure | 1.58 × 10⁻^3^ | 1.00× 10⁻⁴ |
| Macrophage Pathogen Clearance | 1.02 × 10⁻^2^ | 2.00× 10⁻⁴ |
| Liver Cancer Recurrence Signature (Down) | 1.89 × 10⁻^3^ | 2.00× 10⁻⁴ |
| TGF-β1 Signaling (Temporal, Down) | 8.40 × 10⁻^3^ | 3.00× 10⁻⁴ |
| ROS Response in Sepsis | 3.56 × 10⁻^3^ | 3.00× 10⁻⁴ |
| FASLG-RIPK1 Necroptosis Signaling | 2.45 × 10⁻^3^ | 3.00× 10⁻⁴ |
| Regulated Necrosis | 2.73 × 10⁻^3^ | 3.00× 10⁻⁴ |
| HGF Signaling (AKT-Independent) | 2.62 × 10⁻^3^ | 4.00× 10⁻⁴ |
| ERCC6-Mediated DNA Repair (Down) | 2.13 × 10⁻^3^ | 5.00× 10⁻⁴ |
